# Vitamins, Vascular Health and Disease

**DOI:** 10.3390/nu17182955

**Published:** 2025-09-15

**Authors:** George Ayoub

**Affiliations:** Psychology Department, Santa Barbara City College, Santa Barbara, CA 93109, USA; neuro@sbcc.edu

**Keywords:** folate, homocysteine, folate receptor autoantibody, endothelium, lymphedema, dementia, cognitive decline, autism, ASD

## Abstract

Vascular health relies on the proper function of endothelial cells, which regulate vascular tone, blood fluidity, and barrier integrity. Endothelial dysfunction, often aggravated by inadequate vitamin absorption, contributes to a spectrum of clinical disorders, including cardiovascular disease, cerebrovascular disease, peripheral artery disease, age-related macular degeneration, lymphedema, and chronic venous insufficiency. B-group vitamins (especially folate, or vitamin B9), along with vitamins B12, B6, C, D, and E, are essential in maintaining endothelial function, supporting DNA synthesis, regulating methylation, enhancing cellular repair, mitigating oxidative stress and inflammatory signaling, and curtailing vascular damage. Folate is noted for its central function in one-carbon metabolism and in converting homocysteine to methionine, thereby reducing vascular toxicity. We cover natural dietary sources of folate, synthetic folic acid, and the biologically active forms 5-methyl-(6S)-tetrahydrofolate (L-5-MTHF, L-methylfolate) and 5-formyl-(6S)-tetrahydrofolate (levoleucovorin). Therapeutic strategies to address vascular health and prevent hyperhomocysteinemia in order to preclude follow-on disorders include targeted vitamin supplementation, dietary improvements to ensure a sufficient intake of bioavailable nutrient forms, and, in certain clinical contexts, the use of active L-methylfolate or levoleucovorin (a drug product) to bypass metabolic conversion issues. These evidence-based interventions aim to restore endothelial homeostasis, slow disease progression, and improve patient outcomes across a variety of disorders linked to poor vascular health.

## 1. Vitamins in Vascular Health

Vascular health depends on the integrity and function of endothelial cells, which line the interior surface of blood vessels. In degenerative disorders (including disorders as varied as age-related macular degeneration or lymphedema), a diminished absorption of these vitamins leads to poorer vascular health, which exacerbates the disorder [1,2]. Vitamins, particularly B-group vitamins, as well as vitamins C, D, and E, play crucial roles in maintaining endothelial health, supporting cellular functions such as DNA synthesis, methylation, cell repair, and the modulation of oxidative stress. This section focuses on the vitamins essential for vascular health, with a special emphasis on vitamin B9 (folate), its forms and bioavailability, and the consequences of deficiency or insufficiency.

### 1.1. Vitamins Essential for Endothelial Health: Folate

Folate (vitamin B9) is essential for one-carbon metabolism, which supports DNA and RNA synthesis, facilitating cell division and repair [3,4,5]. Folate in one-carbon metabolism also supports methylation reactions, which regulate genes, as well as amino acid metabolism [4].

Vitamins B9 and B12 are also involved in the conversion of homocysteine to methionine. Reducing homocysteine lowers its toxicity, which can otherwise harm blood vessels [3,6].

**Figure 1:**
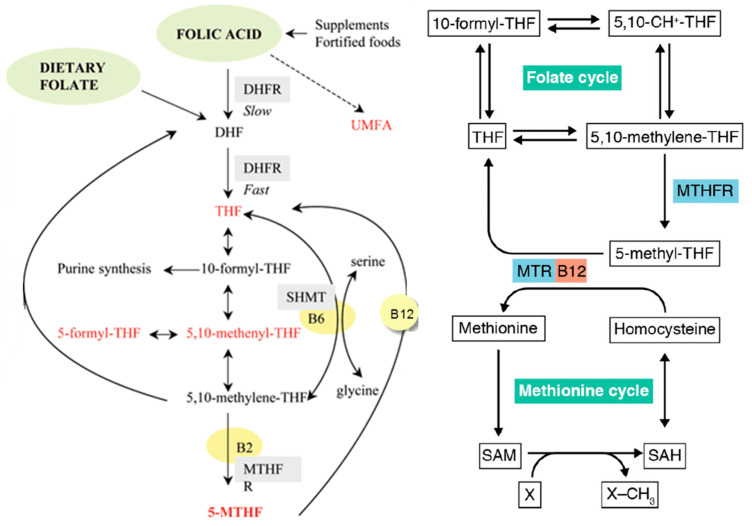
Metabolism of vitamin B9. Left panel shows the typical starting point of dietary folate (present in many foods) or synthetic folic acid (used as a supplement). Folic acid needs to be reduced to di-hydro folate in the gut and the liver, a slow process, before it can be converted to the cellular active version methylfolate (5-MTHF). Right panel shows the role of folate, along with that of B6 and B12, in the metabolism of homocysteine to methionine, preventing homocysteine accumulation, which damages blood vessels [7,8].

There are two forms of dietary folate. The natural form is found in foods (leafy greens, legumes, and liver). It is reduced but requires conversion in the body to the active form utilized by cells [3,5]. The synthetic, more stable form is oxidized and is used in supplements and food fortification. It is better absorbed (~85% bioavailability) than food folate (~50%) but must first be reduced before it can be converted to the active form 5-methyl-(6S)-tetrahydrofolate L-5-MTHF (or L-methylfolate) in the liver and digestive tract [3,5]. Figure 1 shows the pathways of dietary folate and folic acid and their conversion to L-methylfolate, which is the biologically active form [6]. While supplements typically contain synthetic folic acid due to its long-term stability, some use L-methylfolate.

One variation in supplements is whether the active form L-methylfolate is used or the racemic version, which includes the D-isomer in equal amounts. If the supplement is a racemic mix, then the dosing needed is twice that of the active form L-methylfolate. This is true for L-methylfolate, as well as for other reduced forms of folate, such as folinic acid (leucovorin), where the active form is levofolinic acid (levoleucovorin). L-methylfolate is relatively commonly available now, while leucovorin/levoleucovorin are used in approved drug products. In this paper, we use the term methylfolate to refer to the active form L-methylfolate throughout. We note that the full terminology is L-5-methyl-(6S)-tetrahydrofolate (L-5-MTHF) and that the term L-methylfolate is more common, but they refer to the same molecule. We note that, in some referenced publications, different abbreviations such as L-5-MTHF have been used. In clinical practice, it is always best to use the L-isomer of methylfolate or folinic acid (leucovorin) in treatment, as there is no dilution from an equal amount of inactive D-isomer.

#### 1.1.1. Steps in the Folate Pathway

Dietary folates (mainly polyglutamated forms) are deconjugated to monoglutamate forms in the small intestine and absorbed via specific transporters (e.g., proton-coupled folate transporter). Once in the bloodstream, folate is transported to tissues and taken up by cells through reduced folate carriers (RFCs) and folate receptors [5,9].

Inside cells, folate is reduced to dihydrofolate (DHF) and then to tetrahydrofolate (THF) by the enzyme dihydrofolate reductase (DHFR). THF acts as a carrier of one-carbon units in various oxidation states, forming derivatives such as 5,10-methylene-THF, 5-methyl-THF, and 10-formyl-THF [5,10].

THF derivatives participate in purine and thymidylate (dTMP) synthesis (for DNA/RNA); methionine regeneration from homocysteine (via 5-methyl-THF and methionine synthase); and methylation reactions (via S-adenosylmethionine, SAM) [5].

Folate metabolism occurs in both the cytosol and mitochondria, with specific enzymes and folate forms in each compartment. Folate metabolism is tightly regulated, and cells possess repair mechanisms for damaged folate molecules [10,11].

#### 1.1.2. Folate Deficiency/Insufficiency and Vascular Dysfunction

Folate deficiency or insufficiency leads to impaired DNA synthesis, megaloblastic anemia, and elevated homocysteine, and it increases the risk of vascular dysfunction, including atherosclerosis, stroke, and degenerative diseases [3,4]. Elevated homocysteine due to low folate impairs endothelial function and increases oxidative stress, a key factor in vascular disease [3,6].

Supplementation with folic acid or L-methylfolate can improve endothelial function, especially in those with cardiovascular risk or established disease, by increasing nitric oxide (NO) bioavailability and reducing oxidative stress [3,6,12]. Mandatory folic acid fortification in the USA has reduced the rates of neural tube defects and improved population folate status, but concerns remain about unmetabolized folic acid (UMFA) with high supplementation, as it may interfere with cellular access or the use of L-methylfolate [4,5].

### 1.2. Vitamins Essential for Endothelial Health: Pyridoxine and Cobalamin

Vitamin B6 (Pyridoxine) and B12 (Cobalamin) are both cofactors in homocysteine metabolism, working synergistically with folate to convert homocysteine to methionine [3,5]. Vitamin B6 acts as a coenzyme in the transsulfuration pathway, converting homocysteine to cystathionine and then to cysteine. Adequate B6 prevents hyperhomocysteinemia, thereby supporting endothelial function [3]. Additionally, B6 modulates the expression of adhesion molecules and inflammatory cytokines, protecting against endothelial activation and subclinical vascular inflammation [13].

Vitamin B12, in concert with folate, is essential for the remethylation of homocysteine to methionine. Insufficiency exacerbates homocysteine elevation and related vascular threats [14]. Vitamin B12, along with folate, is also necessary for myelin synthesis and repair. For this reason, it is a promising treatment for neuropathic pain, as it may promote myelination and increase nerve regeneration [15].

Both vitamins are readily absorbed from dietary and supplemental sources. They both support DNA synthesis and repair [4]. Absorption can be impaired in common conditions, including aging and gastrointestinal disorders [5]. Additionally, deficiencies can lead to elevated homocysteine, which creates an increased cardiovascular risk [16].

### 1.3. Vitamins Essential for Endothelial Health: Ascorbic Acid

Vitamin C (Ascorbic Acid) is a powerful antioxidant. It neutralizes reactive oxygen species, thus reducing oxidative stress-induced dysfunction. It is active in the regeneration of nitric oxide, which enhances vasodilation, thus reducing hypertension risk, by facilitating NO bioavailability. Vitamin C protects endothelial cells from oxidative damage. It supports collagen synthesis, which is important for the integrity of the vascular lining [3,17].

Vitamin C is highly bioavailable in fruits and vegetables. It is water-soluble and rapidly absorbed [3].

### 1.4. Vitamins Essential for Endothelial Health: Vitamin D

Vitamin D receptors are expressed in endothelial cells, and activation suppresses pro-inflammatory gene expression while enhancing anti-inflammatory signals. It thus modulates inflammation and immune function in the endothelium, supporting vascular resilience [17,18,19,20,21]. A vitamin D deficiency reduces endothelial conductance and increases arterial stiffness [22].

Vitamin D influences the renin–angiotensin–aldosterone system, modulating blood pressure and thus supporting vascular tone via blood pressure regulation [19]. A recent meta-analysis found an association of CVD with low serum vitamin D, with supplementation reducing CVD [20].

Vitamin D is produced in the skin via sunlight exposure. It is also available in fortified foods and supplements [3,17].

### 1.5. Vitamins Essential for Endothelial Health: Tocopherol and Vitamin K2

Vitamin E (tocopherol), especially alpha-tocopherol, is a lipid-soluble antioxidant in cell membranes, protecting endothelial cells from lipid peroxidation. It reduces LDL oxidation and plaque formation and may modulate inflammation and platelet aggregation in blood vessels [3,18].

Vitamin E is absorbed with dietary fats. It is found in nuts, seeds, and vegetable oils [3].

Vitamin K2 helps to regulate soft tissue stiffness by activating an anti-calcific protein. The prevalence of vitamin K deficiency in the USA is high. Because vitamin K2 reduces arterial stiffness, it thus plays a role in reducing CVD [23].

### 1.6. Public Health Aspects

#### 1.6.1. Nutritional Sufficiency

Population studies underscore the prevalence of suboptimal vitamin intake and its correlation with atherosclerotic disease progression and adverse vascular outcomes. Surveys continue to show that, many individuals, including in developed nations, fall short of optimal vitamin intake, especially B9, B12, D, and C [18,24,25]. Treatment of these efficiencies could ideally be addressed with cardiovascular-healthy diets [26], and supplementation may be considered in cases of diminished absorption capacity or a genetic or immune condition limiting the bioavailability of these vitamins.

#### 1.6.2. Genetic Polymorphisms

Polymorphisms in genes encoding enzymes for folate and homocysteine metabolism such as MTHFR increase the need for bioavailable forms of folate (such as L-methylfolate or a drug product containing levoleucovorin). These gene variants may exacerbate deficiencies and vascular risk. In these cases, direct use of L-methylfolate (or levoleucovorin) is beneficial [4,27].

The existence of polymorphisms often raises concerns as to whether there may be variations in the efficacy or negative effects of treatment with a replacement for folate. Because leucovorin/levoleucovorin has been used in cancer therapy for many decades, including in young children, there is ample evidence that, even with much higher doses than discussed here, the administration of leucovorin rescue (referred to as a rescue because it rescues children from the cancer medication methotrexate, which depletes vitamin B9) does not have a negative impact, and the positive effect is present in all ethnic groups [28].

#### 1.6.3. Supplementation and Disease Prevention

Mandatory folic acid fortification has lowered neural tube defect rates. This supplementation must be balanced against issues in those unable to efficiently convert folic acid to its bioactive form. There is increasing evidence that non-deficient individuals and those with gene variants may be negatively impacted by folic acid supplementation [12,18].

Vitamins are indispensable for vascular health through their diverse roles in endothelial cell function, regulation of oxidative and inflammatory processes, and homocysteine metabolism. Timely identification and correction of deficiencies or insufficiencies, targeted supplementation in at-risk individuals, and a foundation of nutrient-rich dietary habits are all strategies with significant potential to reduce vascular disease burden and promote healthy aging.

## 2. Folate Sources, Absorption, and Bioavailability

Vitamin B9, commonly known as folate, is an essential water-soluble B vitamin required for DNA synthesis, amino acid metabolism, and key methylation reactions.

Folate exists both in natural forms found in many food products and as a synthetic compound, folic acid, used in supplementation and food fortification. The natural forms are reduced in form, and folic acid is oxidized, making it stable for long periods of time. Understanding natural and synthetic sources, comparative bioavailability, and the impact of genetic variation on folate metabolism is critical for optimizing health and preventing deficiency-related conditions [4,25,29,30].

### 2.1. Folate from Foods

Folate occurs naturally in several foods. Food products with the highest folate concentrations are shown in Table 1. Among these, spinach, liver, asparagus, and Brussels sprouts are particularly folate-rich [27,30]. The dominant forms of folate in foods are polyglutamated tetrahydrofolates (such as L-methylfolate), which are critical for metabolic functions. The glutamate residues are removed in the gut to provide the active form methylfolate used by cells [31].

Folic acid is a mono-glutamate, and it is fully oxidized and stable. It is chemically distinct from natural food folate in that it does not require deconjugation or release from plant matrices. Fortified foods and supplements provide reliable, high bioavailability forms of folic acid, compensating for losses from natural sources and helping reduce deficiency rates [30,32,33]. Folic acid is present in fortified grains and dietary supplements.

Another form of folate is found in medical foods. These are products that contain L-methylfolate (L-5-MTHF), a bioactive form used in certain clinical situations. Leucovorin/levoleucovorin, bioactive forms used in cancer therapy to replace folate depleted by chemotherapy and used as a treatment to alleviate communication symptoms in autism [34], are classified as drug products. Both are available in the L-form, and, in the past, they were often available in a racemic mix of the L- and D-isomers. In the racemic case, 50% is usable, so the dosages needed are twice that of the L-isomers.

### 2.2. Bioavailability and Absorption

The bioavailability of naturally occurring folate is inherently variable and generally limited compared to that of synthetic folic acid. Typical estimates are that natural food folate is about 50–60% as bioavailable as folic acid consumed with a meal [5,29,35].

Three factors affect natural folate bioavailability. These are that folate can be tightly bound within plant cell structures; that natural folate is polyglutamated, which requires enzymatic deconjugation before absorption; and that folate is susceptible to heat, light, and oxidation, so losses can occur during cooking and storage [5,29,35].

Emerging research suggests that, depending on the food and preparation, bioavailability may range from roughly 44% to 80%, with a median estimate of around 65% as compared to synthetic folic acid [29,35,36]. Labeling of folate in food products includes an indication of the dietary folate equivalent (DFE), where 1 DFE corresponds to 0.6 mg of folic acid or L-methylfolate. This bioavailability variation, along with incomplete liberation from the food matrix and sensitivity to food processing, is a key reason why public health campaigns emphasize folic acid fortification and why this fortification has successfully raised population folate status and reduced the incidence of neural tube defects [5,30].

### 2.3. Genetic Influences on Folate Metabolism

Several common genetic polymorphisms influence folate absorption, metabolism, and the body’s methylation capacity.

MTHFR (Methylenetetrahydrofolate Reductase) gene *C677T* and *A1298C* polymorphisms affect the conversion of folic acid and food folates to the bioactive methylfolate form. This impacts homocysteine levels and increases susceptibility to neural tube defects and ASD in certain populations [37,38,39,40]. Individuals with these variants may have higher homocysteine levels and may benefit from supplementation with L-methylfolate rather than folic acid [1,7,41].

*MTR*, *MTRR*, *FOLH1*, *RFC1*, and *SHMT* genes. These variants can influence the absorption, tissue distribution, and utilization of folate, with downstream effects on DNA methylation and health outcomes [37,38,39,40].

DHFR (dihydrofolate reductase). Variability in DHFR activity can affect the reduction of folic acid to tetrahydrofolate, influencing how efficiently synthetic folic acid is utilized. Excess folic acid may accumulate in individuals with low DHFR activity [42].

### 2.4. Folate Transport into the Brain

Folate must cross the blood–brain barrier (BBB) to support neurological function. This process is mediated by at least two specific transport proteins. Folate receptor alpha (FRα) is essential for high-affinity transportation of folate across the choroid plexus into the cerebrospinal fluid and brain tissue. Mutations in the FOLR1 gene can cause cerebral folate deficiency (CFD), leading to neurological symptoms [43].

The reduced folate carrier (RFC, *SLC19A1*) facilitates folate entry into various tissues, including the brain, but because it does not use active transport, it can only replicate plasma folate concentrations in the brain. Cerebral folate levels are 2–5 times greater in the brain than in blood, with the youngest children having the highest (5×) cerebral folate levels, and the multiplier diminishes through childhood/adolescence to 2×. Genetic variations in the RFC gene can influence folate delivery to the central nervous system. Recent research also implicates the vitamin D receptor (VDR) in the regulation of folate transport across the BBB, especially in certain neurological diseases [44,45].

Disruption of these transport mechanisms, whether by genetic mutations, autoantibodies, or disease, can result in cerebral folate deficiency, a low brain folate level, even when blood folate status appears normal. Treatment with lecovorin/levoleucovorin can sometimes bypass these defects and restore brain folate levels [45].

These genetic factors help explain the differences between individuals in folate requirements; the varied responses to supplementation; and the risk of conditions linked to both plasma folate deficiency and cerebral folate deficiency, including birth defects, cardiovascular disease, and autism spectrum disorder [37,38,39,40].

Research continues to elucidate the interaction between genetic variation, environmental exposures, and folate-dependent pathways, pointing to a future with more personalized recommendations for folate intake [46].

## 3. Cardiovascular Health

Folate improves endothelial function both by lowering homocysteine levels and through homocysteine-independent mechanisms. Evidence from human clinical studies demonstrates that folate can enhance vascular reactivity and may reduce cardiovascular disease (CVD) events, particularly in at-risk populations with low baseline folate or elevated homocysteine levels. The cardiovascular benefit of folate is dose-dependent, and excessive supplementation, especially with folic acid, may pose risks.

Other vitamins, notably B6, B12, C, D, E, and K, play complementary roles in promoting vascular health through antioxidative, anti-inflammatory, and structural vessel support mechanisms.

### 3.1. Folate and Endothelial Function

Folate plays a critical role in one-carbon metabolism, particularly in the remethylation of homocysteine to methionine, as described above. Elevated homocysteine is recognized as an independent risk factor for cardiovascular disease (CVD). Mechanistically, high homocysteine levels can promote endothelial dysfunction via increased oxidative stress, impaired nitric oxide (NO) bioavailability, and direct endothelial injury. By facilitating the conversion of homocysteine to methionine, folate supplementation lowers plasma homocysteine concentrations and may limit these deleterious vascular effects [6,12,47,48].

Moreover, folate exerts endothelial benefits independent of lower homocysteine levels. Experimental studies have demonstrated that folate in the form of folic acid and its bioactive metabolite, L-methylfolate, can directly enhance endothelial function, possibly by reducing intracellular superoxide availability and improving NO production in endothelial cells [6,12]. This provides a plausible explanation for observed improvements in endothelial-dependent vasodilation with folate, even when reductions in homocysteine are modest or absent.

Endothelial dysfunction is a central feature in the pathogenesis of atherosclerosis, preceding plaque formation and influencing the progression of established disease. Improvements in endothelial function, assessed via flow-mediated dilation (FMD), correspond to a reduced cardiovascular risk [12,49]. Both hyperhomocysteinemia and suboptimal folate status have been linked epidemiologically and mechanistically to an increased cardiovascular risk. We note the exception to this published over a decade ago that did not find a link between large-vessel disease and elevated homocysteine levels [50]. The work cited in this paper has shown that, whether that is the case, an elevated homocysteine level is linked to small-vessel disease and that this elevated homocysteine level can be reduced (and capillary diffusion restored) with L-methylfolate supplementation.

### 3.2. Evidence for Improved Blood Flow

Multiple studies have provided evidence that folate, along with an adequate intake of other B vitamins such as B6 and B12, improves vascular reactivity and blood flow. In animal models, folate supplementation has reversed endothelial dysfunction caused by hyperhomocysteinemia [3].

In humans, several randomized controlled trials and observational studies have shown that high-dose folic acid (5–10 mg/day) improves FMD and overall endothelial function in patients both with and without hyperhomocysteinemia [6,12,49,51]. Notably, the effect on vascular health often occurs even before major changes in homocysteine levels are detected, indicating alternative protective mechanisms such as enhanced antioxidant defense and direct effects on NO production.

Beyond folate, vitamins C, E, D, and K—through their antioxidant properties, blood pressure regulation, and support of blood vessel health—contribute to better circulation and may reduce atherosclerosis and peripheral vascular disease risk [2,52].

### 3.3. Clinical Evidence

Trials in patients with coronary artery disease (CAD) demonstrate that folic acid supplementation (5 mg for 6 weeks) increases plasma folate levels and improves FMD, a measure of endothelial function, even when the reduction in homocysteine is moderate [6,12,49]. Meta-analyses suggest that folic acid supplementation is associated with a modest reduction in overall cardiovascular events and stroke risk, particularly in individuals with a low baseline folate status or significant homocysteine elevation. The most pronounced benefits are seen in primary rather than secondary prevention [53].

While moderate folate intake is associated with reduced cardiovascular mortality, some data suggest that excessive folic acid supplementation may not continue to provide incremental benefits and could even have potential adverse effects at the highest intake levels [54,55,56]. This may be due to the limits of how much folic acid can be converted to bioavailable folate each day. It is estimated that up to 400 µg of folic acid is converted per day [57]. When folic acid intake is above 400 µg, it remains in the blood as unmetabolized folic acid (UMFA). This may be a unique problem for humans, as the conversion in rats is 80 times as much as that in humans [57]. There is increasing evidence that UMFA reduces the availability of folate to cells [58,59], making excess folic acid supplementation problematic [60].

Additionally, large randomized trials in selected populations (e.g., chronic kidney disease) have not consistently demonstrated reduced cardiovascular events after long-term folate supplementation, emphasizing the importance of individual risk, baseline folate status, and other confounding factors [61].

The outcomes of the published trials are summarized in Table 2.

The therapeutic impact for CVD is that, in cases with elevated homocysteine, folate (at doses listed in Table 2) improves outcomes. But, if homocysteine levels are not elevated, there may be minimal impact, indicating that plasma homocysteine is an essential biomarker in determining a treatment plan. Toxicity does not appear to be a concern [26], unless a patient with elevated homocysteine levels also has high plasma folic acid levels. In such cases, folic acid should not be used in treatment, and, instead, a reduced form of folate (folinic acid or L-methylfolate) should be used. The guidance here is that, in cases of elevated homocysteine, it would be beneficial to monitor plasma folate and to also screen for vitamin B12 deficiency, which could be masked by the elevated homocysteine. In cases with elevated homocysteine, supplementation with L-methylfolate would be advised, along with monitoring for and treating any diminished B12. Folic acid could be considered for supplementation but it poses a risk due to UMFA [60].

## 4. Peripheral Circulation, Lymphedema, and Glymphatic Function

Folate plays a multifaceted role in circulatory health, improving endothelial function, supporting nitric oxide production, and potentially enhancing both blood and lymphatic flow. Clinical evidence suggests benefits in peripheral artery disease and lymphedema, with promising implications for lymphatic function. While the impact of folate on the glymphatic system remains speculative, its established vascular effects provide a rationale for future investigation into its role in brain waste clearance and neurodegenerative disease prevention [62,63].

### 4.1. Folate and Peripheral Circulation

Folate has been shown to improve endothelial function in the peripheral circulation, with effects that extend beyond its ability to lower homocysteine levels [62]. High-dose folic acid acutely lowers blood pressure and enhances vasodilator-stimulated blood flow in patients with coronary artery disease, likely by increasing nitric oxide bioavailability [3,63]. This is achieved through the enzymatic regeneration of tetrahydrobiopterin, an essential cofactor for nitric oxide synthase, thereby supporting nitric oxide production and vascular dilation [62,63].

Supplemental folic acid can also prevent endothelial dysfunction and nitrate tolerance induced by continuous nitroglycerin therapy, further underscoring its role in maintaining nitric oxide synthase function and vascular health [3,62]. These vascular benefits are observed in both coronary and peripheral arteries, suggesting a broad impact on the systemic circulation [62,63,64].

### 4.2. Folate and Lymphedema

Folate’s influence extends to the lymphatic system, where it may promote lymphangiogenesis, the formation of new lymphatic vessels, and improve lymphatic flow. Evidence from a human study supports the role of folate in enhancing lymphatic circulation [2]. This clinical case reported successful use of L-methylfolate in managing lymphatic congestion and limb swelling in a patient with primary lymphedema [2]. This suggests that folate supplementation could be a promising adjunct in the management of lymphedema, potentially by improving lymphatic vessel function and reducing interstitial fluid accumulation [2,65,66]. Such treatment would have fewer secondary effects than the proposed pharmacological use of endothelial growth factors [67].

### 4.3. Glymphatic System

The glymphatic system is a recently described waste clearance pathway in the brain, analogous to the peripheral lymphatic system. It facilitates the removal of metabolic waste products and is thought to play a role in neurodegenerative diseases such as dementia [68,69,70]. While direct evidence is limited, it has been speculated that folate could influence glymphatic circulation through mechanisms similar to those observed in peripheral and lymphatic vessels [2]. If folate enhances nitric oxide production and vascular health, it may also support the function of perivascular spaces and astroglial water channels that drive glymphatic flow [71].

A diminished glymphatic system has been hypothesized to contribute to the accumulation of neurotoxic waste products in the brain, potentially increasing the risk of dementia [72,73]. If folate improves glymphatic clearance [74], it could offer neuroprotective benefits. While this has been shown in the retina [1], trials showing an effect across the blood–brain barrier are needed. While this concept remains speculative, it warrants further research due to the substantial impact that it can have on an aging population. Studies to confirm this conjecture are being developed, including the evaluation of L-methylfolate intervention as an adjunct therapy in an ongoing study on cognitive decline.

## 5. Retinal Vascularization and Retinal Disease

Folate deficiency leads to hyperhomocysteinemia, which damages retinal vascular endothelial cells through oxidative stress, inflammation, and barrier breakdown. Proper folate status and transport are essential for homocysteine detoxification; endothelial protection; and the maintenance of retinal vascular integrity in eye diseases such as diabetic retinopathy, retinal vascular occlusions, glaucoma, and age-related macular degeneration (AMD) [75,76,77,78]. In all four of these disorders, the capillaries are sensitive to hyperhomocysteinemia, resulting in a reduction in vascular perfusion and an increase in retinal venous pressure [1,41,79].

### 5.1. Retinal Hyperhomocysteine and Vascular Dysfunction

As noted above, folate is a key dietary determinant of plasma homocysteine. Adequate folate enables the remethylation of homocysteine to methionine, keeping homocysteine levels low. Folate deficiency is the most common cause of elevated homocysteine (hyperhomocysteinemia) [75,76,78]. Elevated homocysteine exerts direct toxic effects on retinal vascular endothelial cells, contributing to the pathogenesis of retinal vascular diseases such as retinal vascular occlusions, diabetic retinopathy, glaucoma, and age-related macular degeneration. Mechanistically, homocysteine disrupts endothelial cell function by inducing oxidative stress, reducing tight junction protein expression, increasing vascular permeability, and promoting inflammation, which collectively compromise the blood–retinal barrier and retinal vascular integrity [76,77,80].

Experimental models show that high homocysteine levels lead to a loss of retinal ganglion cells and the thinning of retinal layers, effects that can be partially reversed with folate supplementation [78]. This suggests that folate’s protective role extends beyond lowering homocysteine levels, possibly involving direct support of endothelial cell health and antioxidant capacity.

The efficacy of folate in protecting retinal vasculature depends on its transport into retinal cells, primarily in the form of L-methylfolate. Efficient folate transport is crucial for maintaining the integrity of the inner blood–retinal barrier and resistance to ischemia. Impaired transport or retinal folate deficiency can occur even with normal serum folate levels, leading to increased local homocysteine and vascular dysfunction [75,81]. Folate, particularly L-methylfolate, also supports the production of nitric oxide (NO), a vasodilator that maintains retinal blood flow. Impaired folate availability can lead to vasoconstriction and exacerbate ischemic injury in the retina [75].

### 5.2. Retinal Oxidative Stress and Antioxidants

Oxidative stress is a central mechanism by which elevated homocysteine (hyperhomocysteinemia) induces retinal damage. High homocysteine levels increase the production of reactive oxygen species (ROS) in retinal endothelial and glial cells, overwhelming the eye’s natural antioxidant defenses and leading to cellular dysfunction and tissue injury [77,82,83].

Homocysteine-induced oxidative stress reduces the expression of tight junction proteins in retinal endothelial cells, increasing vascular permeability and contributing to blood–retinal barrier (BRB) breakdown, a hallmark of vision loss in diseases like diabetic retinopathy and age-related macular degeneration [77,84].

Excess ROS trigger inflammatory pathways and promote apoptosis (cell death) of retinal neurons, including retinal ganglion cells, further compromising retinal structure and function [82,84,85]. Elevated homocysteine not only generates ROS but also diminishes antioxidant capacity, as seen by reduced levels of protective enzymes (e.g., superoxide dismutase and glutathione peroxidase) and total antioxidant capacity in affected patients and experimental models [83].

The oxidative environment created by high homocysteine contributes to endothelial toxicity, hypercoagulability, and an increased risk of retinal vascular occlusions such as central retinal vein occlusion [83].

Experimental evidence shows that antioxidant treatment can mitigate some of these effects, reducing ROS formation and restoring barrier function in homocysteine-exposed retinal cells [77]. Thus, oxidative stress is a key mediator of homocysteine-induced retinal damage, driving vascular dysfunction, barrier breakdown, inflammation, and neuronal loss in a range of retinal diseases [77,82,85].

### 5.3. Clinical Cases

Recent studies have proven the efficacy of using medical food levels of B vitamins, using the L-methylfolate form of vitamin B, to treat elevated homocysteine levels in individuals with eye diseases. In each case, the treatment used the medical food Ocufolin, which is a medical food vitamin supply containing different doses of the AREDS2 proven therapy for dry AMD, with L-methylfolate, vitamin B12, and other B vitamins to support endothelial cell health (this medical vitamin perhaps may become referred to as AREDS3 to indicate the addition of vascular support from the B vitamins). In each study, the treatment reduced homocysteine levels and improved retinal perfusion, leading to improved outcomes in each retinal disease.

For diabetic retinopathy, L-methylfolate reduced homocysteine and improved retinal perfusion while also reducing stroke risk, leading to the recommendation to evaluate patients for central nervous system microangiopathy using retinal imaging [86].

In a small case study, glaucomatous conditions were alleviated with similar vitamin treatment. In this study, retinal venous pressure was measured, showing that it was elevated in high-tension glaucoma, as well as in normal-tension glaucoma, and that the medical food therapy reduced this venous pressure, thus improving retinal perfusion and patient outcomes [7,41]. This study revealed that normal-tension glaucoma patients have elevated retinal venous pressure and that the vitamin therapy provides a viable treatment for this challenging-to-treat condition. Retinal venous pressure more accurately measures intraocular conditions that reduce vascular perfusion and lead to disorders such as glaucoma [79].

Age-related macular degeneration (AMD) patients were similarly evaluated for retinal venous pressure, and it was found that Ocufolin vitamin therapy reduced it, as well as reducing homocysteine levels. It was further found that the use of this oral vitamin treatment may reduce the frequency of anti-VEGF ocular injections, which are the standard of care in AMD, with stronger outcomes seen when using vitamin therapy, suggesting that reducing homocysteine and retinal venous pressure, alongside anti-VEGF, may be the most effective treatment of neo-vascular AMD [1]. This promising finding suggests that the use of vitamin therapy should be included as an adjunct with anti-VEGF treatment, as no adverse effects of the vitamin therapy have been found, and, if this first finding is supported by larger trials, then such vitamin therapy may become a standard of care included for treating neo-vascular AMD. Using it as adjunct therapy with anti-VEGF may allow for an extended time between anti-VEGF treatments under close monitoring.

## 6. Neurodegenerative Disorders: Dementia and Cognitive Decline

Folate deficiency or insufficiency, along with elevated plasma homocysteine levels, has emerged as a significant modifiable risk factor for neurodegenerative disorders, particularly dementia and cognitive decline [87]. This section evaluates data from epidemiological, clinical, and mechanistic studies to elucidate folate’s role in brain health and neurodegeneration.

Current evidence supports folate’s role in mitigating neurodegeneration through homocysteine regulation, endothelial cell maintenance, DNA repair, and anti-inflammatory pathways. While population-level fortification has reduced severe deficiency, suboptimal folate status remains a risk factor for cognitive decline. Targeted supplementation may benefit deficient individuals, but universal recommendations require further stratification by age, genetic factors, and baseline nutrient status.

### 6.1. Neurodegenerative Disease and Folate

Folate deficiency is a significant modifiable risk factor for neurodegenerative disorders, particularly dementia and cognitive decline [87]. This report synthesizes evidence from epidemiological, clinical, and mechanistic studies to elucidate folate’s role in brain health and neurodegeneration. As explained above, folate deficiency elevates homocysteine, a neurotoxic amino acid associated with vascular damage and neuronal apoptosis. Hyperhomocysteinemia disrupts endothelial function and increases oxidative stress, contributing to Alzheimer’s pathology and cerebrovascular disease [88,89,90].

Folate is critical for nucleotide synthesis and methylation. Experimental studies in mice lacking uracil glycosylase demonstrate that folate deficiency exacerbates uracil misincorporation into DNA, leading to hippocampal neuron death and reduced brain-derived neurotrophic factor (BDNF) [88]. Impaired DNA repair mechanisms may accelerate age-related cognitive decline.

Low folate also correlates with elevated pro-inflammatory cytokines (e.g., IL-6 and TNF-α) and reduced glutathione, a key antioxidant. These factors synergistically promote neuronal damage and inhibit hippocampal neurogenesis [88,91].

### 6.2. Folate Deficiency Linked to Cognitive Decline

There is extensive clinical evidence of the impact of folate deficiency on cognitive decline. A longitudinal study of 3140 older Irish adults found that folate levels < 5 ng/mL predicted accelerated global cognitive decline, while levels < 9 ng/mL impaired episodic memory [92]. Note that serum folate levels between 4 and 9 ng/mL are considered borderline, while serum levels under 4 are considered low [30]. An earlier study (Sacramento Area Latino Study) found that folate was directly associated with cognitive function and inversely associated with dementia [93].

A Korean study found that low–normal serum folate levels (1.5–5.9 ng/mL) increased the risk of cognitive impairment, finding that, in their 4-year period, the dementia risk was significantly higher for people with those folate levels [94]. An Israeli study found that serum folate levels in an older population of less than 4.4 ng/mL increased dementia risk by 68% and increased the all-cause mortality risk by a factor of three [89]. This compares with the Irish and Latino studies, where data showed that, post-fortification, there was a persistent association between low–normal folate and cognitive impairment, suggesting that optimal thresholds may exceed current deficiency criteria [92,93].

Low serum and red cell folate levels are consistently associated with an increased risk of cognitive decline, Alzheimer’s disease, and vascular dementia in elderly populations. Prospective studies have shown that folate or vitamin B12 deficiency can double the risk of developing Alzheimer’s disease [95].

Data from large cohorts indicate that higher dietary folate intake is associated with reduced odds of cognitive impairment, with a linear relationship observed between total folate intake and lower risk of global cognitive decline [96].

### 6.3. Folate Supplementation Slows Cognitive Decline

Folate supplementation has been extensively studied for its potential to improve cognitive function in elderly populations at risk of cognitive decline, particularly those with mild cognitive impairment or low baseline folate status.

In a 12-month clinical trial of 180 patients with mild cognitive impairment in China, it was found that folic acid supplementation of 400 μg/day improved global cognition test scores on multiple assessments, significantly raised serum folate and lowered homocysteine, and reduced IL-6/TNF-α by 30% [91]. In the US, 400 μg/day is the recommended folic acid supplement amount [30].

A high-dose supplementation (5 mg/day) in Japanese elders with cognitive impairment or Alzheimer’s disease significantly improved cognition, as measured with the Mini-Mental State Exam (MMSE), and this was correlated with homocysteine reduction [97]. A meta-analysis evaluated a nutritional intervention with folate and observed that it plays an important role in slowing the progression of cognitive decline but that there is a need to make the optimal supplementation levels clear. Additionally, the authors observed that homocysteine levels may be a useful biomarker [98]. In their comparisons of the studies, they found seven studies on folate, four on folate and B12, and ten on B6 and B12. All the studies that included folate found a benefit, reducing cognitive impairment and biomarkers associated with impairment, while the studies without folate found no cognitive improvements [98].

A 2024 meta-analysis of seven randomized controlled trials involving over 1100 older adults (mean age 65–80) with MCI found that folic acid supplementation resulted in significant improvements in several cognitive domains, including Full-Scale IQ, arithmetic, information, digit span, and block design scores. The intervention also reduced inflammatory cytokines and homocysteine levels, both of which are implicated in neurodegeneration [99].

Another comprehensive review of 51 studies (including 23 meta-analyzed) concluded that folate-based B vitamin supplementation has a significant overall positive effect on cognitive function in older adults, particularly in regions without mandatory folic acid food fortification. In countries with fortification policies, additional supplementation did not yield significant cognitive benefits, likely due to an already adequate folate status in the population [100].

In a 6-month trial in China (a country without folic acid fortification), elderly individuals with MCI who received 400 µg/day of folic acid showed significant improvements in cognitive performance (Full-Scale IQ, digit span, and block design) compared to controls. These improvements were accompanied by increased serum folate and vitamin B12 and reduced homocysteine levels [101].

Historical studies and open-label trials have also reported that folic acid supplementation (often at 5 mg/day) improved mood, initiative, alertness, and cognitive function in folate-deficient elderly patients, with some patients showing marked functional recovery [95].

To date, the published data refer to the use of folic acid in addressing cognitive impairment [90,102]. Especially striking from the Smith and Refsum study of the impact of B vitamins in slowing brain atrophy is that, in individuals with elevated homocysteine levels, those receiving B vitamin therapy in the blinded study had little to no brain atrophy, while those on placebo had substantial atrophy, as seen in their Figure 11 [102]. Additionally, they documented with an MRI scan that, while placebo treatment showed a 5.2% atrophy rate in gray matter over 2 years, the B vitamin treatment group had a 0.6% reduction in the same period [102].

Given that some fraction of the population may have a limited effect from folic acid, studies using L-methylfolate would be welcome, as this folate should have a similar impact for all people. We are planning such a study to identify whether L-methyfolate may have a more universal effect in slowing cognitive decline.

### 6.4. Efficacy of Folate in Slowing Cognitive Decline

The cognitive benefits of folate supplementation are most pronounced in populations with low baseline folate or without mandatory food fortification. In countries with widespread folic acid fortification, further supplementation may not confer additional cognitive benefits for most older adults [100].

Individuals with MCI or documented folate deficiency are more likely to benefit from supplementation than cognitively healthy or folate-replete individuals [99,101]. Excessive folate supplementation in certain high-risk groups (e.g., those with cardiovascular disease) may have adverse effects, and supplementation should be used cautiously in the presence of vitamin B12 deficiency or epilepsy [95,96,103].

Folate supplementation can improve cognitive function in at-risk elderly populations, especially those with mild cognitive impairment or low baseline folate status, and in regions without mandatory folic acid fortification. The greatest benefits are observed in individuals with documented deficiency or elevated homocysteine. Supplementation is less likely to be effective in populations with adequate folate levels due to food fortification. Additionally, folate absorption and utilization entail adequate vitamin B12 levels, so a B12 deficiency can impact efficacy or other diseases [104]. Further high-quality trials are needed to define optimal dosing and identify subgroups most likely to benefit [99,100,101]. In this regard, there is potential for machine learning systems to glean evidence from large databases, such as the UK Biobank, to identify nutrients that increase and those that decrease dementia risk [105].

### 6.5. Risks of Excessive Folate in Disease

Folate is essential for cardiovascular and neurological health, largely due to its role in homocysteine metabolism and DNA repair. While adequate folate intake is beneficial, excessive folate supplementation in older adults with CVD may pose certain risks.

The relationship between folate intake and cardiovascular disease (CVD) in older adults is complex, with emerging evidence suggesting potential risks associated with excessive folate consumption in this population. Excessive folate intake risks for older adults with CVD include masking B12 deficiency, potential increases in mortality risk, and attenuation of cognitive benefits. For these reasons, supplementation needs to be tailored, and high-dose folic acid needs to be avoided. These risks may be unique to folic acid, suggesting that routine use of methylfolate may mitigate risk while conferring the cognitive benefit. These findings highlight the need for personalized folate recommendations in older adults with CVD, contrasting with general population guidelines [96,106]. Table 3 summarizes the high-dose folic acid risks.

#### 6.5.1. Excess Folic Acid and Increased Mortality Risk

Excess folic acid is linked to increased CVD mortality in multiple studies. While modest folate intake showed improvement in long-term survival, excess folic acid in CVD patients increased mortality [54,107]. High red blood cell folate levels (>1080 nmol/L) are linked to a 32% higher risk of CVD mortality and a 25% increased risk of all-cause mortality in older adults with preexisting CVD [96,106].

These findings of a benefit with moderate folate levels and danger from high levels reveal a U-shaped relationship for folate. Low (<476 nmol/L) and high red blood cell folate levels correlate with elevated CVD mortality in patients with type 2 diabetes [96].

These data show that moderate dietary folate (400–600 μg/day) reduced mortality risk, while high supplemental folic acid (>1000 μg/day) increased mortality risk by 18–24% [53,96].

#### 6.5.2. Excess Folic Acid and Attenuated Cognitive Benefits

As detailed above, folate supplementation can improve cognition in deficient individuals. However, excessive intake may not confer additional benefit and could even attenuate protective effects, especially in those with CVD or diabetes.

While red blood cell folate normally protects against cognitive impairment, this protective effect disappears entirely in CVD patients [96]. CVD may exacerbate insulin resistance and cerebral microcirculation damage, counteracting folate’s neuroprotective benefits [96].

#### 6.5.3. Pathways of Harm from Excess Folic Acid

There are a number of proposed mechanisms for why excess folic acid can be problematic, especially in CVD patients.

- Folate oversaturation in fortified populations: Over 50% of US CVD patients already meet the recommended folate intake, yet 25% still use supplements [96].
- Interaction with CVD pathophysiology: High folate may accelerate atherosclerosis progression in established CVD through poorly understood mechanisms [106].
- Homocysteine paradox: While folate lowers homocysteine, excessive supplementation in CVD patients shows no mortality benefit despite homocysteine reduction [108].
- Masking vitamin B12 deficiency: High folate intake can mask hematological signs of vitamin B12 deficiency, which is common in older adults. This can allow neurological damage from B12 deficiency to progress undetected, potentially leading to irreversible cognitive and neurological impairment [59].
- Potential for unmetabolized folic acid (UMFA): A high intake of synthetic folic acid (from supplements/fortified foods) can lead to unmetabolized folic acid in the bloodstream, which has been linked to impaired immune function and possibly an increased cancer risk [58,109]. This risk from unmetabolized folic acid, when found during pregnancy and lactation, may also be associated with an increased risk of neurodevelopmental disorders such as autism [110,111], as well as with a tripling in the rate of gestational diabetes [112]. Given that excessive folic acid supplementation is the cause of UMFA, there is reason to reconsider the amount supplemented. In the US, supplementation provides about 200 µg on average, though some individuals have much higher levels, greatly exceeding the 400 µg daily conversion in the body. Switching to L-methylfolate would reduce this risk.

#### 6.5.4. Guidelines for Folate Supplementation in CVD Patients

The data support avoiding the use of high-dose folic acid supplements in older adults with CVD history. High-dose folic acid supplements are over 400 μg/day. The published at-risk threshold is for red blood cell folate levels over 900 nmol/L in CVD patients, which warrants caution [96].

The recommendations to avoid this are to prioritize dietary folate (leafy greens and legumes) over synthetic folic acid [96,106]. Additionally, monitoring of red blood cell folate levels in CVD patients taking B-complex vitamins is essential [96].

Given that it is the use of folic acid, which is the oxidized version of folate, that is problematic, if dietary folate (which is a reduced folate) is insufficient to meet the folate need, the use of other reduced forms of folate, namely, L-methylfolate, would be advised. In this regard, high-dose folinic acid (prescription name, leucovorin) has been used for over 75 years as a prescription drug product for vitamin B9 replacement (in chemotherapy). No cardiotoxicity has been observed with this high-dose leucovorin, with the exception that leucovorin in combination with 5-fluorouracil had the same cardiotoxicity (3%) as 5-fluorouracil alone [113].

The guidelines for folate supplementation in CVD patients can be summarized as follows:

- Avoid high-dose folic acid supplements (>400 μg/day) in older adults with CVD unless prescribed for a specific deficiency.
- Monitor vitamin B12 status in older adults taking folic acid, especially those at risk of deficiency.
- Prioritize dietary sources of folate (leafy greens and legumes) over high-dose supplements, unless medically indicated.
- When supplementation is warranted, prioritize the use of methylfolate.
- Personalize supplementation based on individual risk factors, comorbidities, and baseline folate/B12 status.
- Consider high-dose treatment with a drug product containing leucovorin or levoleucovring based on specific diagnostic tests (such as genetic tests and the FRAA test).

## 7. Neurodevelopmental Disorders: Autism Spectrum Disorder

Autism spectrum disorder is a multifactorial disorder with origins in the fetal period [114]. Folate is essential for early brain development, and maternal deficiency is associated with an increased risk of ASD in offspring. Recent research has explored the relationship between maternal folate status and the risk of autism spectrum disorder (ASD), as well as the therapeutic potential of folate treatment in children with ASD. High-dose leucovorin (folinic acid) supplementation has shown promise in improving the core symptoms of ASD, particularly in children with mitochondrial dysfunction or folate receptor autoantibodies [34,115,116,117]. The role of folate in methylation and epigenetic regulation provides a plausible biological mechanism for these effects. Future research focusing on personalized interventions based on genetic and metabolic profiles should benefit treatment options. Using levoleucovorin will benefit further studies by eliminating the inactive D-isomer and including only the fully active L-isomer (levoleucovorin).

### 7.1. Folate’s Role in Brain Formation During Pregnancy

Folate is vital for the closure of the neural tube during embryogenesis, which occurs within the first month of pregnancy. Inadequate maternal folate intake is a well-known risk factor for neural tube defects such as spina bifida and anencephaly. Beyond neural tube defects, folate is crucial for early brain development, influencing neuronal proliferation, migration, and synaptogenesis [118,119].

Rodent models have demonstrated that maternal folate deficiency leads to impaired neurogenesis; an abnormal brain structure; and behavioral changes reminiscent of ASD, such as reduced social interaction and increased repetitive behaviors [120].

Epidemiological studies have linked low maternal folate levels or a lack of periconceptional folic acid supplementation to increased ASD risk in offspring. A large Norwegian cohort study found that maternal use of folic acid supplements around conception was associated with a lower risk of autistic disorder in children [121]. Similarly, a meta-analysis concluded that maternal folic acid supplementation significantly reduced the risk of ASD [122].

Folate is a key donor of methyl groups for DNA methylation, an epigenetic mechanism that regulates gene expression. Disruptions in methylation pathways have been implicated in ASD. Children with ASD often exhibit abnormal DNA methylation patterns and altered expression of genes involved in synaptic function and neurodevelopment [123]. Low folate increases homocysteine levels, which is neurotoxic and can impair neuronal migration, synapse formation, and myelination. Elevated homocysteine during gestation has been associated with an increased risk of neurodevelopmental disorders in offspring.

Folate supplementation may help correct these epigenetic disruptions. For instance, animal studies show that maternal folate supplementation can reverse methylation abnormalities and behavioral deficits in offspring exposed to environmental risk factors for ASD [120].

Maternal folate deficiency during early brain development increases autism risk through multiple mechanisms: impaired DNA synthesis and repair, epigenetic dysregulation, and elevated neurotoxic homocysteine. Both animal and human studies support the protective effect of adequate maternal folate against ASD. Ensuring sufficient maternal folate intake before and during early pregnancy is a key public health strategy to reduce the risk of autism and optimize neurodevelopmental outcomes.

Recent clinical trials have investigated the effects of high-dose leucovorin (a bioactive form of folate) in children with ASD, particularly those with mitochondrial dysfunction or folate receptor autoantibodies [124]. Multiple trials in which ASD children received high-dose leucovorin (2 mg/kg/day, up to 50 mg/day) for 12 weeks have been conducted. In each, the treatment group showed significant improvements in verbal communication, especially in children with folate receptor autoantibodies [34,115,116].

### 7.2. Maternal Folate Deficiency and Autism Risk

Decreased access to folate during gestation can be due to low maternal folate levels, genetic variants, or autoimmune disorders [46,125]. Table 4 summarizes the impact of maternal folate deficiency and its link to autism. Insufficient folate conveys multiple risks for neurodevelopmental disorders.

#### 7.2.1. Impaired DNA Synthesis and Neuronal Repair

Folate is essential for DNA synthesis and repair, especially during periods of rapid cell division in early embryonic brain development. Deficiency leads to DNA instability and impaired neuronal repair, potentially causing abnormal brain structure and function, key features implicated in autism spectrum disorder (ASD) [126].

#### 7.2.2. Homocysteine Accumulation and Neurotoxicity

Folate regulates homocysteine metabolism. Without adequate folate, homocysteine levels rise. Elevated homocysteine is neurotoxic. It can disrupt neuronal migration, synapse formation, and myelination, all critical for normal brain development. High maternal homocysteine is associated with an increased ASD risk in offspring [127].

#### 7.2.3. Disrupted Methylation and Epigenetic Regulation

Folate provides methyl groups for DNA methylation, a key epigenetic process that controls gene expression during neurodevelopment. Deficiency results in hypomethylation, leading to the abnormal activation or silencing of genes crucial for brain development, synaptic function, and neuronal connectivity. Epigenetic dysregulation is a recognized mechanism in ASD etiology [126].

#### 7.2.4. Altered Neurotransmitter Synthesis

Folate is involved in the synthesis of neurotransmitters such as serotonin, dopamine, and norepinephrine. Deficiency may lead to imbalances in these neurotransmitters, which are often observed in individuals with ASD [128].

#### 7.2.5. Oxidative Stress and Inflammation

Low folate levels increase oxidative stress and susceptibility to neuroinflammation. Both oxidative stress and chronic inflammation are implicated in the pathogenesis of ASD [129,130]. A valuable biomarker that has systematically indicated children that will respond positively to leucovorin treatment is the presence of the folate receptor autoantibody (FRAA) in blood plasma. FRAA is produced by the immune system in response to a dietary product [131]. In multiple clinical trials, ASD children with FRAA showed improvement in symptoms when treated with daily leucovorin (up to 2 mg/kg body weight) [34,115,116]. Larger-scale controlled trials are needed to determine whether this can become standard practice of care, and planning for such a trial is underway.

### 7.3. Critical Periods for Folate and Autism

Research consistently identifies early pregnancy, particularly the periconceptional period (just before and shortly after conception) and the first trimester, as the most critical window for folate intake to reduce autism risk in offspring. Multiple large cohort and case–control studies demonstrate that folic acid supplementation starting before conception and continuing through early pregnancy is associated with a significantly lower risk of autism spectrum disorder (ASD) in children [121,132,133,134].

The Norwegian Mother and Child Cohort Study found a 40–45% reduction in autistic disorder risk among women who took folic acid supplements from four weeks before to eight weeks after conception [132]. Similarly, a meta-analysis of published studies where folic acid was supplemented during pregnancy (but not prior) found a significant reduction in the risk of ASD [122].

When leucovorin was supplemented (at 7.5 mg/day) periconceptionally and throughout gestation in two women expressing folate receptor autoantibodies, and with prior ASD births, the children were neurotypical at three years of age [135]. This indicates that during maternal periconception and gestation periods, a more universal treatment for mothers with elevated risk may be leucovorin rather than folic acid.

The first month of pregnancy, when the neural tube forms and closes, is especially sensitive to folate status [127]. Supplementation during this period appears to be crucial for both preventing neural tube defects and reducing ASD risk.

Continuous folic acid supplementation from the periconceptional through to the prenatal period may offer the greatest protective effect to reduce ASD risk [132,133,135]. Women who missed supplementation during both periods had the highest risk of having a child with ASD [132,133].

Some evidence suggests a U-shaped relationship: both low and excessively high maternal folate levels may increase ASD risk, while moderate, recommended intake is optimal [132]. This may be unique to folic acid supplementation, as a study with 7.5 mg/day of leucovorin did not exhibit any high folate risk [135].

#### 7.3.1. Insights from Folate Supplementation in Reducing Risk of Autism

An important critical period for folate intake to prevent autism is from at least one month prior to conception to the first trimester of pregnancy. Supplementation during this window is strongly associated with a reduced risk of ASD in offspring [121,132,133,134].

The overall observations are as follows:

- Begin Supplementation Before Conception: Start at least one month before trying to conceive and continue through the first trimester of pregnancy [121,132,133,134].
- Maintain Adequate Intake: 400 micrograms (mcg) of folic acid daily is the widely recommended dose for women planning pregnancy and during early gestation [127,133].
- Continue Through Early Pregnancy: Ensure consistent intake through the first 2–3 months of pregnancy, the period of neural tube and early brain development [127,133].

Maternal folate is crucial for neurodevelopment during critical pregnancy windows due to its central role in several fundamental biological processes required for healthy brain formation and function in the fetus. We recommend trials to confirm and expand this, and we are actively engaged with proposing studies to accomplish this.

#### 7.3.2. DNA Synthesis and Cellular Proliferation

Folate is essential for DNA synthesis and repair, supporting the rapid cell division and growth that occur during early embryonic development. This is especially important for neural stem cell proliferation and differentiation, which lay the foundation for the brain’s structure and complexity [119,136,137]. The roles of folate in early development include the following:

- Neural Tube Closure. During the first month of pregnancy, folate is critical for the closure of the neural tube—a process that, if disrupted, results in severe neural tube defects such as spina bifida and anencephaly. This is why periconceptional folic acid supplementation is universally recommended to women planning pregnancy [119,136,137,138].
- Epigenetic Regulation and Gene Expression. Folate acts as a methyl donor in one-carbon metabolism, which is required for DNA methylation. Proper methylation regulates the gene expression patterns necessary for normal brain development. Disrupted methylation due to folate deficiency can lead to long-lasting neurodevelopmental and cognitive changes in offspring [119,136,137].
- Neurotransmitter and Phospholipid Synthesis. Folate is involved in the synthesis of key neurotransmitters (serotonin, dopamine, norepinephrine, and acetylcholine) and phospholipids, both of which are vital for neuronal signaling, connectivity, and myelination [119,136,137].
- Prevention of Cell Death (Apoptosis). Adequate maternal folate reduces apoptosis (programmed cell death) in developing brain regions, ensuring proper formation of neural circuits [137].
- Maintenance of Healthy Homocysteine Levels. Folate helps maintain low homocysteine concentrations. Elevated homocysteine is neurotoxic and has been linked to impaired neurodevelopment [119,136].

Human studies, while sometimes inconsistent due to methodological differences, generally support that adequate maternal folate, especially during the periconceptional period and first trimester, is associated with better neurodevelopmental and cognitive outcomes in children [119,136,137,139]. This is summarized in Table 5.

Maternal folate is vital during critical pregnancy windows because it underpins DNA synthesis, gene regulation, neurotransmitter production, and neural tube closure, all of which are foundational for healthy fetal brain development [119,136].

#### 7.3.3. Insights from Folate Supplementation in Reducing Risk of Autism

- Periconceptional and first-trimester folate intake are most strongly associated with reduced ASD risk and better neurodevelopmental outcomes [121,132,133,134,135].
- Very high maternal folate/B12 levels late in pregnancy may be associated with an increased ASD risk, suggesting that moderation is important [132].
- Genetic factors (e.g., MTHFR polymorphisms) can modify the protective effect of folate [132,134].
- Plasma/whole-blood folate levels alone are less predictive than supplement use, possibly due to timing and individual metabolism [132].
- The use of leucovorin during the periconceptional period and throughout gestation may mitigate the risk associated with gene variants and maternal FRAA [135].

### 7.4. Leucovorin Clinical Trials

Leucovorin (5-formyl-tetrahydrofolate, folinic acid) is a bioactive form of folate that bypasses certain metabolic blocks, including those caused by folate receptor autoantibodies or mitochondrial dysfunction, both of which are more prevalent in many children presenting with autism spectrum disorder. Folate receptor alpha autoantibodies are present in approximately 70% of ASD children [34]. Mitochondrial dysfunction is estimated to occur in up to 5% of children with ASD, and it can impair folate transport into the brain, potentially worsening neurodevelopmental symptoms [124].

Clinical trials using leucovorin have been completed in multiple countries, with similar findings in all [34,115,116,117,140]. All trials were double-blind, placebo-controlled trials with children under 15. The intervention used was leucovorin at 2 mg/kg/day (up to 50 mg/day for 12 weeks). All found significant improvement in verbal communication in the majority (but not all) of children presenting with folate receptor autoantibodies and little to no effect in children testing negative for the antibody. Leucovorin was well-tolerated, with mild side effects of irritability and insomnia being rare and manageable.

The findings indicate that leucovorin can lead to clinically meaningful improvements in core ASD symptoms, especially language and communication, in children with mitochondrial dysfunction or cerebral folate deficiency [34,115,116,117,140].

The benefits are most pronounced in subgroups with biomarkers indicating impaired folate transport or metabolism, as measured by the presence of the folate receptor autoantibody.

Leucovorin is effective due to three features:

- Bypasses Blocked Folate Transport. Leucovorin is rapidly metabolized to L-methylfolate, which can cross the blood–brain barrier via alternative transporters, even when folate receptor alpha is blocked by autoantibodies or the individual has a genetic polymorphism reducing active transport.
- Supports Mitochondrial Function. Leucovorin may enhance mitochondrial energy production, reduce oxidative stress, and improve neuronal metabolism.
- Restores Methylation. As a methyl donor, it supports DNA methylation and neurotransmitter synthesis, processes often disrupted in ASD.

A limitation of leucovorin treatment is that not all children respond. The most significant benefits are seen in children with folate receptor autoantibodies and/or with mitochondrial dysfunction. Because most studies are short term (12–24 weeks), long-term efficacy and safety require further research.

High-dose leucovorin is an effective and generally well-tolerated intervention for improving core symptoms, particularly language and communication, in ASD children who have mitochondrial dysfunction or folate receptor autoantibodies. The strongest evidence comes from randomized controlled trials and open-label studies. The benefits are most pronounced in biomarker-positive subgroups. Further research is needed to optimize dosing and duration and to identify all responsive phenotypes [34,115,116,117,140].

## 8. Conclusions

Current evidence demonstrates that deficiencies or insufficiencies in key vitamins, including folate (B9) and vitamins B6 and B12, C, D, and E, play a pivotal role in the pathogenesis of endothelial dysfunction, arterial stiffness, and subsequent development of a range of vascular and degenerative disorders. Restoration of vitamin status, especially with bioactive forms and targeted supplementation, improves endothelial function and microvascular health, underscoring the therapeutic potential of correcting deficiencies. Table 6 summarizes the disorders discussed in this review and which vitamin deficiencies or excesses are linked to that disorder.

**Figure 2:**
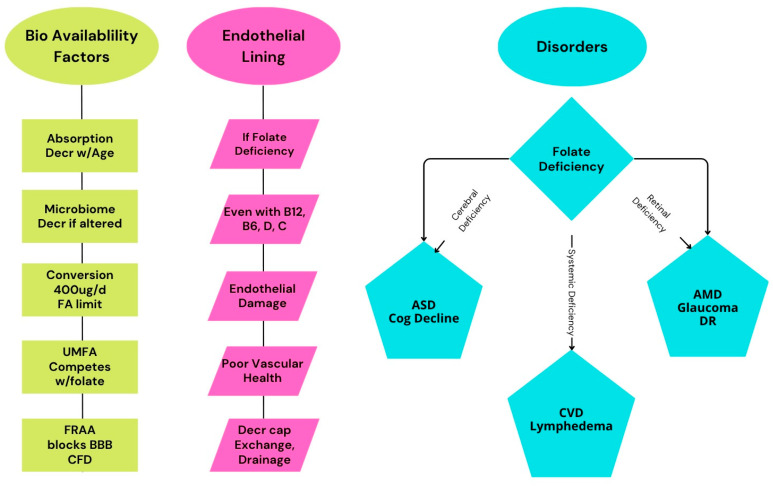
The factors involved in disorders connected with folate deficiencies. The disorders are divided into three categories depending on whether the folate deficiency is systemic, retinal, or cerebral. Systemic disorders include cardiovascular disease and lymphedema. Retinal disorders include AMD, glaucoma, and diabetic retinopathy. Cerebral disorders can be neurodevelopmental, such as autism (ASD), or neurodegenerative, such as dementia or cognitive decline. The driving forces for each category is a reduction in endothelial health, which results in diminished capillary exchange and reduced drainage from the perfused tissue. The capillaries can be systemic blood or lymphatic capillaries, retinal capillaries, or cerebral glymphatic capillaries. The aspects that influence endothelial health are the availability of bioactive folate, along with vitamin B12. Folate availability can be diminished in five ways. There is a reduction in dietary folate absorption in the gut with age; there can be alterations in the gut microbiome that reduce the chemical conversion of ingested folate; excess levels of folic acid can be consumed; excess unmetabolized folic acid enters the blood and competes with folate, reducing its access to cells; and, in a portion of the population, the presence of folate receptor autoantibodies (FRAAs) prevents sufficient folate from entering the cerebral cavity. In most cases, homocysteine provides a reliable biomarker, as small elevations indicate a decline in vascular health. Treatment typically includes reducing folic acid supplementation to under 400 μg/day; confirming the availability of vitamin B12; increasing natural folate through foods and/or leucovorin or L-methylfolate supplementation and, in more severe cases, a drug product containing leucovorin or levoleucovorin; and possibly restoring microbiome diversity.

Clinically, poor vascular health resulting from impaired vitamin metabolism or absorption can manifest as cerebrovascular disease, lymphedema, cognitive decline, age-related macular degeneration, glaucoma, and diabetic microvascular complications. The mechanisms driving these associations involve hyperhomocysteinemia, increased oxidative stress, impaired nitric oxide signaling, defective collagen synthesis, and heightened inflammation—all modifiable via nutritional intervention and supplementation, as summarized in Figure 2.

Despite strong observational and mechanistic studies supporting the link between vitamin deficiency and vascular risk, interventional trials have produced mixed results, particularly for cardiovascular disease events. Nevertheless, normalization of vitamin levels, particularly vitamin D and homocysteine-lowering B vitamins (with the most effective forms of such B vitamins), can improve vascular function metrics and reduce risk factors. These findings suggest that clinical strategies prioritizing adequate vitamin intake, tailored supplementation, and the use of bioavailable forms may enhance patient outcomes and help prevent the progression of disorders rooted in poor vascular health. Additionally, neurodegenerative disorders such as autism are impacted by cerebral folate deficiency and show benefit from treatment with natural folate in the form of leucovorin.

One of the overarching points throughout this review has been the variation in folate source, whether natural, from foods and L-methylfolate supplements; synthetic, from folic acid fortified in grain products (the most recent CDC list can be found in [141]); or taken as oral tablets (which usually contain folic acid rather than L-methylfolate). In countries such as the US, where foods are supplemented with folic acid, it is estimated that the average vitamin B9 intake per day for adults is 450–600 µg (the lower value is for women, and the higher value is for men) and that half of it is in the form of folic acid. With the recommended daily need for folates being 400 µg, this puts many in a situation of excess vitamin B9. But the disparity of vitamin intake, even in the US, is quite high, with estimates of some individuals having twice these averages and others having only 20% of them. Given that a percentage of the overall population has a genetic polymorphism or an immune response making folic acid not sufficiently available, as well as the fact that unmetabolized folic acid (UMFA) interferes with folate availability to cells and the brain, we likely have a case of problems caused by the extremes, with many impacted by too much folic acid and another group having an insufficient folate intake, impacting the disorders summarized in Table 6. It may be recommended that healthy adults obtain their B vitamins from food sources and that supplements be reserved for those with a higher risk of disease due to their genetics or immune state or due to declining absorption often seen past middle age, along with women seeking pregnancy (though there is a caution that excess folic acid in pregnancy may be an important risk factor for gestational diabetes [112]). Perhaps most needing supplements would be best advised to use natural forms to avoid the risk of UMFA. This is an area where more research is needed, including comparisons of the incidence of the disorders discussed here and the levels of natural and synthetic folate seen in people in each country. Recognizing that many disorders have a link to a deficiency of folate or an excess of folic acid, that a vitamin B12 insufficiency can be masked by UMFA, and that measurement of homocysteine (and UMFA as needed) may be the best biomarker for disorder risk is a path that can benefit many.

Overall, we suggest consideration of folic acid supplementation policies given that such supplementation may be maladaptive for a significant fraction of the population, specifically those with folate metabolism polymorphisms or immune production of FRAA. It may be that, in the current environment, a large cohort of individuals who are susceptible to UMFA may be inadvertently consuming excess folic acid in prepared foods. Consideration of the lowering of folic acid supplementation may be called for. In clinical practice, we recommend routine screening for hyperhomocysteinemia and, when found, to test for vitamin B12 deficiency, to test for plasma folic acid, and to treat with L-methylfolate sufficient to decrease homocysteine levels. With severe folate insufficiencies such as those seen in autism, treatment with drug products containing leucovorin or levoleucovorin is warranted. Such treatment is typically based on a diagnostic test for cerebral folate deficiency. In several published studies, this has been successfully addressed with the daily provision of several mg of L-methylfolate, along with vitamins B12, B6, and D, with no adverse effect.

Optimizing vascular health requires a multifaceted approach: correcting vitamin deficiencies, understanding their molecular effects on endothelial and smooth muscle cells, and integrating nutritional therapy with standard clinical care. Further trials are warranted to define optimal intervention protocols and outcomes for vitamin-based therapies in the prevention and management of degenerative vascular diseases.

## Figures and Tables

**Table 1:** Key food sources of folate.

Food Product	Example Foods
Dark green leafy vegetables	Spinach, kale, collard greens, and romaine lettuce
Legumes	Beans, lentils, and peas
Fruits	Oranges, avocados, bananas, and melons
Nuts and seeds	Peanuts and sunflower seeds
Other vegetables	Asparagus, Brussels sprouts, broccoli, and beets
Animal products	Liver and eggs (liver being especially high)
Whole grains	Fortified and unfortified whole grain products

Sources: [4,25,27,30].

**Table 2:** Evidence for folate in cardiovascular conditions.

Outcome	Dose/Duration/Ref.	Population	Notes
Improved FMD *, lowered homocysteine	5 mg folic acid, 6 wks [6,12]	CAD * patients	Benefits independent of homocysteine
Improved FMD *	5–10 mg, 6–8 wks [49]	Hyperhomocysteinemia	
Reduced plaque progression	2.5–5 mg, 4 y [49]	Premature atherosclerosis	With B6, B12
J-shaped risk curve; benefit to modest folate intake	Various (food/suppl) [54]	US adults at CVD * risk	High intake possibly adverse
↓ CVD * events (RR~0.96), ↓ stroke (RR~0.90)	Various [53]	General and high-risk adults	Greater benefit with low baseline
No effect on CVD risk	Folic acid, 2 y [61]	CVD * patients	

* FMD = flow-mediated dilation, CAD = coronary artery disease, CVD = cardiovascular disease, ↓ = decrease.

**Table 3:** Excessive folate risks.

Risk Factor	Evidence
Masking B12 deficiency	Well-established, especially in elderly
Increased CVD/all-cause mortality	U-shaped association in epidemiological studies
Attenuated cognitive benefit	Seen in CVD/diabetes populations with high folate intake
Unmetabolized folic acid	Potential immune/cancer risk, more research needed

Excessive folate refers to folate in the folic acid form. Apart from the masking of a B12 deficiency, use of a reduced form of folate such as L-methylfolate may mitigate these risks.

**Table 4:** Effect of maternal folate deficiency as a contributor to autism risk.

Mechanism	Effect of Folate Deficiency	Link to Autism Risk
DNA synthesis and repair	Impaired neuronal development	Structural/functional brain defects
Homocysteine metabolism	Homocysteine accumulation, neurotoxicity	Disrupted neurodevelopment
DNA methylation (epigenetics)	Hypomethylation, gene dysregulation	Aberrant gene expression in brain
Neurotransmitter synthesis	Imbalanced serotonin/ dopamine/norepinephrine	ASD-related behavioral changes
Oxidative stress/ inflammation	Increased neuronal damage	Neurodevelopmental risk

**Table 5:** Factors in maternal folate intake and related offspring outcomes.

Factor	Timing/Context	Outcome/Association	Supporting Evidence
Maternal leucovorin in women with FRAA	Periconceptional (before and just after conception), through gestation	ASD risk reduced; small study of 2 cases, no ASD outcomes	[135]
Maternal folic acid supplementation	Periconceptional (before and just after conception), 1st trimester	ASD risk decreased by 50% or more	[121,132,133,134]
Maternal folic acid supplementation	No supplementation during pregnancy	Potential increased ASD risk; fetal brain may be sensitive to excess micronutrients	[133]
Maternal plasma/whole-blood folate concentration	Early pregnancy	Mixed findings; some studies show no direct association with autistic traits	[132]
Maternal plasma folate and B12 (very high levels)	At delivery, 3rd trimester	Potential increased ASD risk; fetal brain may be sensitive to excess micronutrients	[132]
Maternal MTHFR gene variant (C677T)	With low folic acid intake	Stronger association between low maternal folate and increased ASD risk	[132,134]
Prenatal multivitamin use (with folic acid)	Preconception and 1st month of pregnancy	Reduced ASD diagnosis and symptom severity in children	[121,132]
Lack of prenatal vitamin/folic acid use	Preconception and 1st month of pregnancy	Higher risk of ASD and more severe symptoms	[121,132]

**Table 6:** Vitamin deficiencies or excesses implicated in disorders discussed.

Disorder	Folate/B9	B12	B6	C	D
Cardiovascular Disease	Deficiency, Excess†	Deficiency	Deficiency	Deficiency	Deficiency
Cerebrovascular Disease	Deficiency	Deficiency	Deficiency	Deficiency	Deficiency
Peripheral Artery Disease	Deficiency	Deficiency	Deficiency	Deficiency [133]	Deficiency
Lymphedema	Deficiency	Deficiency	Deficiency	Deficiency	Deficiency [132]
Age-Related Macular Degeneration	Deficiency	Deficiency	Deficiency	Deficiency [132]	
Diabetic Retinopathy	Deficiency	Deficiency	Deficiency		
Glaucoma	Deficiency	Deficiency	Deficiency		[121,132]
Dementia/Cognitive Decline	Deficiency, Excess †	Deficiency	Deficiency		[121,132]
Autism Spectrum Disorder	Deficiency, Excess †	Deficiency	Deficiency		

Excess † (B9/folate) indicates that disease risk or adverse effects can be aggravated by excessive folic acid supplementation (seen in CVD, neurodegeneration, and autism risk). Blank cells indicate that evidence for vitamin involvement is limited or absent for that condition.

## Data Availability

No new data were created or analyzed in this study. Data sharing is not applicable to this article.

## Abbreviations

The following abbreviations are used in this manuscript:

L-5-MTHF	5-methyl-(6S)-tetrahydrofolate, L-methylfolate
MTHFR	MTHF reductase
DHFR	Dihydrofolate reductase
CVD	Cardiovascular disease
FMD	Flow-mediated diffusion
CAD	Coronary artery disease
VEGF	Vascular endothelial growth factor
UMFA	Unmetabolized folic acid
DFE	Dietary folate equivalent
CFD	Cerebral folate deficiency
AMD	Age-related macular degeneration
MCI	Mild cognitive impairment
ASD	Autism spectrum disorder
